# Influence of the Storage of *Cistus ladanifer* L. Bales from Mechanised Harvesting on the Essential Oil Yield and Qualitative Composition

**DOI:** 10.3390/molecules26082379

**Published:** 2021-04-19

**Authors:** Irene Mediavilla, María Amparo Blázquez, Alex Ruiz, Luis Saúl Esteban

**Affiliations:** 1 CEDER-CIEMAT, Autovía de Navarra A-15, Salida 56, 42290 Lubia, Soria, Spain; luis.esteban@ciemat.es; 2 Departament de Farmacologia, Facultat de Farmàcia, Universitat de València, Avda. Vicent Andrés Estellés s/n, 46100 Burjasot, Valencia, Spain; amparo.blazquez@uv.es; 3 CHROMESSENCE, C./Pompeu Fabra 25, La Garriga, 05530 Barcelona, Spain; alex.ruiz@chromessence.com

**Keywords:** *Cistus ladanifer* L., distillation, essential oil, mechanised harvesting, pilot plant, rockrose

## Abstract

*Cistus ladanifer* is a Mediterranean native plant from which valuable products, such as essential oil, are obtained. Manual harvesting of the plants in wild shrublands is usual during short periods of time. Their mechanised harvesting could increase the volume of harvested plants and prevent fires, further storage of the plants collected being necessary. The objective of this work is to study the influence of the storage period of mechanically harvested bales on the essential oil yield and qualitative composition. The harvesting trials were carried out with an adapted commercial harvester baler and the storage of the bales was performed indoors during 1–7 days, 15–30 days and 100–120 days. Afterwards, the bales were crushed (30 mm) and distilled in a 30 litre stainless steel still with saturated steam (0.5 bar). The essential oil components were identified by GC-MS and quantified by GC-FID. The storage of mechanically harvested *Cistus ladanifer* does not decrease the oil yield of steam distillation on a pilot scale. However, it leads to differences in the quantitative composition of the essential oils, decreasing the total monoterpene compounds content and increasing that of oxygenated sesquiterpenes, especially when the biomass is stored for 100–120 days, without affecting its qualitative composition.

## 1. Introduction

*Cistus* plants, also known as rockroses, are a Mediterranean native genus of shrubs included in the Cistaceae family. The *Cistus* genus contains 25 different species and most of them are very fragrant, sweet-smelling and secrete essential oils. Some of them, like *Cistus ladanifer* subspecies *ladanifer* L., also secrete labdanum, a sticky exudate covering leaves and young stems [1].

**Figure 1:**
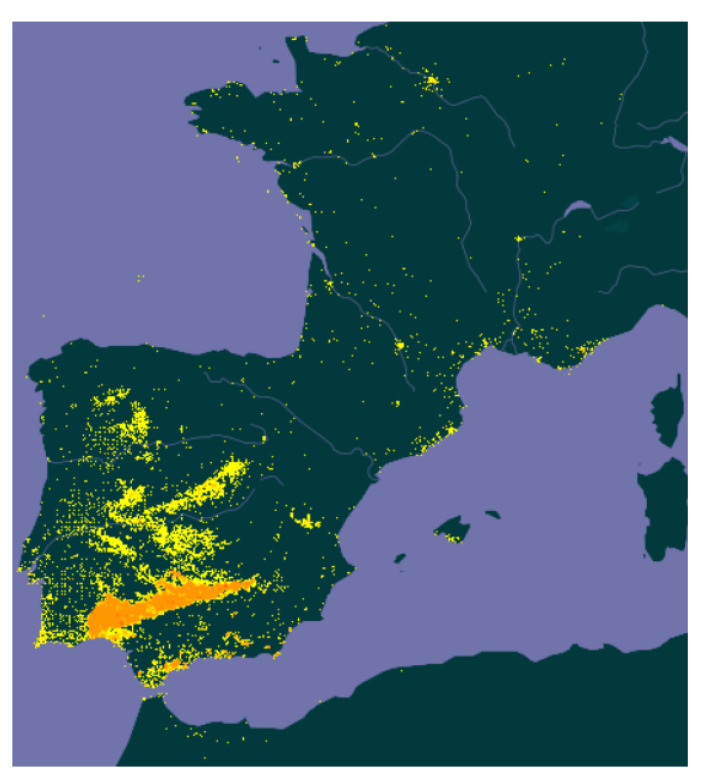
Distribution of *Cistus ladanifer* in Spain and other southern areas according to GBIF (Global Biodiversity Information Facility) [13].

*Cistus ladanifer* grows on sunny, acidic siliceous soils, at altitudes usually below 1000 m and the plant is generally between 1 and 2 m high [2]. In the Mediterranean region, it has dominant nature, with adaptation to extreme environmental factors such as the ability to survive in low hydric and high solar exposition conditions [3,4,5]. Moreover, it is a pyrophyte plant [6,7] and often forms dense scrub communities that produce phytotoxic compounds (likely allelopathic) that may have inhibitory effects on the herbaceous understorey [3,8,9,10,11]. Therefore, *Cistus ladanifer* can eliminate competition and become a colonising species. It usually covers great areas of burnt degraded forests, abandoned pastures and crops in Spain, Portugal, south of France and north of Morocco [3]. The biggest extensions of *Cistus ladanifer* wild shrublands can be found in Spain where, according to recent estimations, the species is present in more than 2 million hectares and is dominant in a little more than 460,000 ha [12] (Figure 1).

Wildland fire is one of the major disturbance factors in Mediterranean ecosystems [7] and shrub clearings, together with other actions, have been shown to be an effective method to control fires and improve the land management of abandoned areas [14]. In this sense, and considering that *Cistus ladanifer* is a fire-prone plant species [6,7], clearings in areas where this plant is dominant and form dense and continuous shrubs could prevent fires and generate substantial amounts of sustainable raw material to obtain high added value products and bioenergy within the biorefinery concept.

Today, the harvesting of *Cistus ladanifer* biomass is concentrated in Spain, with little activity registered in Portugal, France and Morocco. Within Spain, the region of Andalusia is the main producer [15], but there is great potential in other regions such as Extremadura, Castilla La Mancha and Castilla y León (Western and Central regions). Considering the literature [15] and the result of a consultation with the main producer of *Cistus ladanifer* essential oil in Spain according to REACH (Registration, Evaluation and Authorization of Chemicals) [16] (Biolandes), the annual harvest of this biomass is between 10,000 and 20,000 tonnes.

*Cistus ladanifer* secretes a large amount of exudate from its leaves and photosynthetic stems through trichomes. This exudate is characterised by two families of secondary metabolites, flavonoids and diterpenoids [17], and it plays an important ecophysiological role [18] which depends on the concentration of these metabolites, whose synthesis is conditioned not only by environmental factors but also by an intrapopulation variation [17].

Although the typical product obtained from the plant is the labdanum gum, other products can be produced from *Cistus ladanifer*. Labdanum gum can be obtained by immersion of the aerial parts of the plant in warm carbonated water followed by acidification of the medium [19]. This method is widely used in small artisanal devices and in some small processing factories in Southern Spain [20] and currently the total estimated annual production of labdanum gum varies between 200 and 600 t/year (communication from the company Biolandes), with six companies registered in the REACH [16]. If the labdanum gum is distilled, labdanum oil is obtained. On the contrary, if the gum undergoes an alcoholic extraction followed by concentration, labdanum resinoid is produced. The plant exudates can also be extracted using solvents. Therefore, the *Cistus* concrete is produced by solvent extraction of the plant aerial parts and subsequent elimination of the solvent by concentration of the extract, and the *Cistus* absolute is obtained by washing the concrete with ethanol, followed by filtration to eliminate the waxes and concentration of the alcoholic phase [19]. Finally, *Cistus* essential oil and hydrolate (also referred to as hydrosol or floral water), are obtained by hydrodistillation or steam distillation of the leaves and stems. The essential oil yields are very low and the current market prices may exceed 400 €/kg [21]. The global production of essential oil is estimated to be between 1 and 10 t/year, considering the result of a consultation with the only producer registered in the REACH with more than 1 t/year [16] (Biolandes), although higher estimations (50–100 t/year) have been made in the literature consulted [22]. It must also be noted that, although a significant part of the essential oil is produced in small steam distillation plants, the quantity of 50 t/year should require at least an annual plant harvest of more than 50,000 t only for steam distillation, and this is not realistic according to the consulted companies (Biolandes and El Jarpil).

The essential oil has an increasing potential use not only in perfumery and cosmetics industries but also in aromatherapy, pharmaceutical, agricultural and food industries [1,3,23,24], mainly due to its antimicrobial and antioxidant activities.

The most used technique to obtain essential oils on an industrial scale is steam distillation. As far as we know, very few studies on distillation of *Cistus ladanifer* on an industrial scale have been published. Typical yield values between 0.1% and 0.3% have been reported by Ruiz García et al. [21] and some industrial producers consulted (Biolandes and El Jarpil) consider that a common yield value with fresh biomass is close to 0.1%. Experiences carried out in steam distillation pilot plants have also been poorly published, with yield values between 0.01% and 0.04% obtained with 100 kg of plants in a stainless steel distiller of 1100 L during 1.5 h at 0.5 bar [25]. However, there are many studies where the distillation of this plant is performed on a laboratory scale through hydrodistillation by Clevenger’s apparatus [25,26,27,28,29,30], with oil yields between 0.08% and 0.6%.

The low yields obtained during *Cistus ladanifer* distillation in the industry entail the use of large quantities of biomass whose harvesting is usually carried out during a short period of time, from July to October [4]. The plants are currently manually harvested, this being very hard work as it is performed during the hottest days. Teams of harvesters cut the twigs using a sickle, bundle them up and pile them into cars and horse-drawn carts. Since the harvesting period is short, peaks of worker demand can be too high to be covered by the labour market. In this sense, mechanised harvesting could cause considerable improvements in the collection of this biomass, increasing the volume of harvested plants and improving the working conditions. However, the mechanised harvesting of rockrose is a complex matter mainly due to the stickiness of the plant.

In order to solve the aforementioned harvesting issues, the Centre for the Development of Renewable Energies attached to The Centre for Energy, Environmental and Technological Research (CEDER-CIEMAT) has adapted a commercial harvester baler BIOBALER WB55 and performed different harvesting assays in wild shrublands with promising results [31,32,33,34,35].

Taking into account a new framework in which mechanised harvesting is used in a sustainable way and higher harvesting yields are obtained in comparison to manual labour, higher storage periods before the distillation would be needed to carry out an appropriate management of the *Cistus ladanifer* logistics. The mechanised harvesting assays in *Cistus ladanifer* shrubland performed by CEDER-CIEMAT have allowed workers to collect the aboveground plant biomass in round big bale format and store the bales for long periods of time. The effect of the storage of this plant on the steam distillation process has not been reported previously. Therefore, the objective of this work is to study the influence of the storage period of mechanically harvested *Cistus ladanifer* bales on the distillation yield and on the essential oil composition in a pilot scale steam distillation plant.

## 2. Results

### 2.1. Distillation Tests

**Figure 2:**
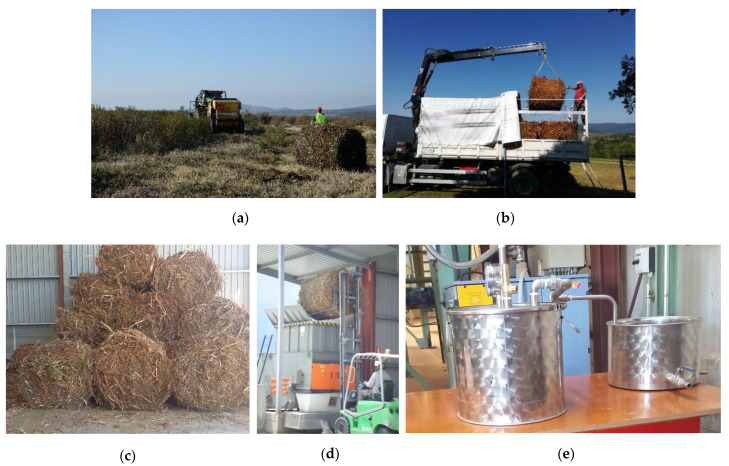
Processes carried out during the pretreatment of the plant material: (**a**) mechanised harvesting of *Cistus ladanifer*, (**b**) transport of the bales, (**c**) indoor storage, (**d**) crushing of the bales and (**e**) distillation of the milled biomass.

*Cistus ladanifer* bales from mechanised harvesting, with an average weight of 359 kg of wet matter per bale and an average moisture content of 30.7%, were stored indoors under good ventilation conditions to prevent mould growth during the storage time. After the storage period, the bales were milled at 30 mm and distilled using the operating conditions shown in Section 4.2. The different processes carried out can be seen in Figure 2.

The yield obtained from essential oil distillation tests, expressed in weight percentage and referred to dry plant, is shown in Table 1.

Within each one of the periods considered (1–7 days, 15–30 days and 100–120 days), the variability between distillations was rather high, as can be noticed in the values of the relative standard deviation (15.5% in the first period, 21.9% in the second period and 13.0% in the third one). It can be considered a normal variability between bales, related to variations in the proportion of young twigs and wood, rather than because of the time that elapses after harvesting.

Although a decreasing trend was observed in the essential oil yield, the statistical analysis of the data obtained showed that this decrease was not significant at the 95.0% confidence level, since *p*-value of the F-test was 0.0911, greater than 0.05.

### 2.2. Essential Oil Composition

The essential oils corresponding to the same storage period were blended and analysed as explained in Section 4.3. *Cistus* oil is one of the most complex essential oils, composed of more than 250 substances of many chemical classes. The main components identified and their quantification using the relative area percentage are shown in Table 2, where it can be observed that monoterpene hydrocarbons and oxygenated sesquiterpenes were the principal phytochemical groups in the essential oils here analysed.

**Figure 3:**
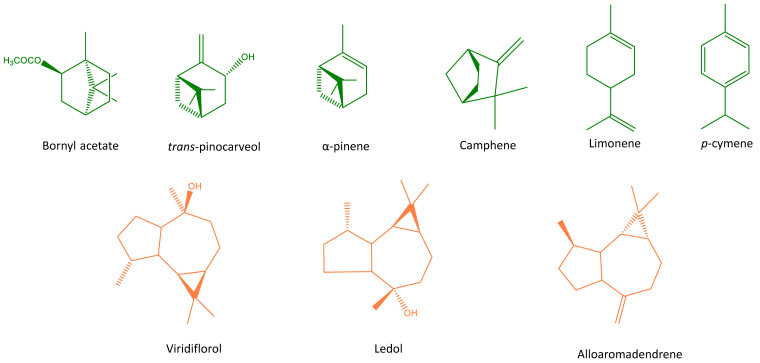
Main compounds of essential oils obtained from stored *Cistus ladanifer.*

Among the monoterpene hydrocarbons identified in the three periods analysed (0–7 days, 15–30 days and 100–120 days, respectively), α-pinene was the main compound (49.65%, 47.42% and 46.67%) followed by camphene (2.60%, 2.49% and 2.56%), limonene (2.08%, 1.81% and 1.52%) and *p*-cymene (1.47%, 1.26% and 1.33%). Considering the oxygenated monoterpenes, bornyl acetate (2.71%, 2.32% and 2.48%), followed by *trans*-pinocarveol (1.56%, 1.59% and 0.99%) can be highlighted. With regard to the sesquiterpene hydrocarbons, alloaromadendrene (1.50%, 1.13% and 1.68%) was the main compound identified. In addition, finally, if the oxygenated sesquiterpenes are considered, viridiflorol (10.03%, 11.98% and 12.50%), followed by ledol (2.85%, 3.41% and 3.51%) were the most abundant compounds. The chemical structures of the abovementioned main compounds are shown in Figure 3.

## 3. Discussion

Regarding the distillation tests, the average essential oil yields are lower than those obtained by other authors who used only manually collected twigs or leaves and hydrodistilled them in a Clevenger apparatus (laboratory scale), with yield values as much as 0.6% [29]. However, they are higher than the values shown by Tavares et al. [25] for the steam distillation in a pilot plant (0.01–0.04%).

Although the decrease in the essential oil yield over the period considered is not statistically significant, a decrease of 12.0% with regard to the fresh plant distillation is observed when the storage is between 15 and 30 days, and a decrease of 30.7% when the storage period is between 100 and 120 days. Considering the literature [37], it could be due to the volatilisation of some compounds during the storage or to the biomass biodegradation caused by physiological deterioration and other factors. The first alternative seems to be a more likely reason if we attend to the reduction in the monoterpenes from 60.92% to 56.50%, and oxygenated monoterpenes, from 10.34% to 8.15%, as can be seen in Table 2. In addition, neither mouldiness nor other visual alterations were observed after the storage, and no temperature increases were detected inside the bales either.

The composition of *Cistus ladanifer* essential oil depends on several factors, including the origin of the plant, the season when the harvest was carried out or the oil extraction method used [1,3,25,26,27]. In this sense, different compositions have been found in literature. According to Ruiz García et al. [21], commercial essential oils usually show high α-pinene content, it being desirable not to exceed 30%, and sometimes using a partial removal of this component. Other major components are also shown, such as *trans*-pinocarveol, with limits between 2.0% and 7.0%, ledene, ranging between 1.0% and 6.0% and viridiflorol, with typical values between 1.5% and 3.5%. Other authors that have analysed commercial *Cistus* essential oils have reported results for Spanish essential oils [27] with α-pinene (48.9–50.0%), camphene (2.4–5.0%), 2,2,6-trimethyl cyclohexanone (1.5–2.0%), bornyl acetate (1.5–3.1%), *trans*-pinocarveol (1.7–2.8%), viridiflorol (1.1–1.7%) and ledene (1.4–3.7%) as the main compounds. The analysis of commercial Portuguese essential oils [25] has shown the same major compounds and different limits, i.e., α-pinene (29.8–59.5%), camphene (2.6–14.7%), 2,2,6- trimethyl cyclohexanone (0.4–3.4%), bornyl acetate (2.1–6.1%), *trans*-pinocarveol (1.8–5.9%), viridiflorol (0.8–1.9%) and ledene (0.3–4.5%). On the other hand, considering literature where parameters regarding plants, harvesting, oil extraction methods and oil analysis are studied, different compositions of the oil have been found. Therefore, very low α-pinene and camphene contents, around 2% and 0.1%, respectively [26,27], *trans*-pinocarveol contents up to 10.9% [26] and viridiflorol contents higher than 15% [26,27,38,39] are reported in some of the samples analysed. On the other hand, especially high globulol values [27], between 3.1% and 5.0%, 1–8 cineole values (19.3%) [38], γ-terpinene (6.1%) [38] and γ-gurjunene (14.6%) [39] are shown in some of the studies.

In this sense, the comparison of the data obtained in the present work with analyses found in literature and market data from industrial scale, where the conditions to obtain the essential oil can be different, shows quantitatively relevant differences, mainly in the content of key sesquiterpene alcohols like viridiflorol, whose content is higher in these experiments than in most of the analyses of commercial and non-commercial oils reported.

The total compounds identified in the essential oils analysed are similar in all three cases (94.39%, 94.15% and 92.30%). Moreover, the biomass origin and the pretreatment and distillation operating conditions remained constant in all the experiments. Consequently, it can be highlighted that when the storage period increases, the percentages of total monoterpene hydrocarbons and total oxygenated monoterpenes tend to decrease while the percentages of total oxygenated sesquiterpenes tend to increase, especially when the samples corresponding to 0–7 days and 100–120 days (14.57% vs. 18.64%) are compared. Taking into account the different compounds, α-pinene, verbenene, limonene and β-phellandrene are the monoterpene hydrocarbons with the higher decrease. Regarding the sesquiterpene hydrocarbons, the change is not remarkable in any of the compounds identified. Finally, within the oxygenated sesquiterpenes, the increase in viridiflorol and ledol can be highlighted. However, it is interesting to note that no qualitative differences were observed in the composition of the essential oil in the different storage periods.

Future research is necessary to study the influence of the density of *Cistus ladanifer* bales on the storage, the essential oil yield and its chemical composition. Similarly, additional research to determine if the storage outdoors, keeping bales dry, produces significant changes in the quantity and quality of the *Cistus* essential oil is planned.

## 4. Materials and Methods

### 4.1. Plant Material

The plant material was obtained with a harvester baler system (BIOBALER WB55, Chesterville, QC, Canada). This equipment, powered by a 154 kW tractor (Valtra T194D, Suolahti, Finland), includes a harvester unit that cuts standing shrubs at an approximate height of 15 cm above the ground, and leads vegetation to a cylindrical baling system that compresses the biomass into round big bales. This machine was acquired in 2015 and tested with different shrub vegetation and terrain conditions in the ENERBIOSCRUB project between 2015 and 2018 [40]. Some tests were carried out in *Cistus laurifolius* shrublands, a species similar to *Cistus ladanifer* but with much less content of sticky exudates [33].

The study was performed on 12.97 ha of abandoned cropland covered by rockrose (*Cistus ladanifer*) in Hiendelaencina—Guadalajara (Spain), at an altitude of 1030 m above sea level in October 2018. The site has cool Mediterranean climate, according to Köppen classification, with an annual average rainfall of 585 mm, average temperature of the warmest month below 22 °C and 69 frost days per year. Soil conditions were similar in the whole area, with gentle slope, low terrain roughness and no stoniness. The area was mainly covered by rockrose shrubs with a mean height of 0.5 m, estimated age between 5 and 10 years and average ground crown cover of 40%.

After the harvesting, the bales were transported to CEDER-CIEMAT. Then, four samples were taken from four different bales and the moisture content of the biomass was analysed following the standard ISO 18134-2:2017 (Solid biofuels—Determination of moisture content—Oven dry method—Part 2: Total moisture—Simplified method). Afterwards, the bales were stored indoors during three different periods: 1–7 days (just harvested), 15–30 days (less than one month from harvest) and 100–120 days (long storage period).

After the storage period, the corresponding bales were crushed to a size of 30 mm by means of a shredder (90 kW, slow rotating single-shaft type, SILMISA, Onil, Spain), just before the distillation tests.

### 4.2. Distillation Tests

Ten water steam distillation tests were performed, each one corresponding to a different storage time: 1, 2, 4, 7, 15, 20, 30, 100, 110 and 120 days. Four consecutive samples of 30 mm milled *Cistus ladanifer* (5 kg each) were distilled for each one of the tests. The essential oil obtained was accumulated and the yield was calculated for the whole distillation process. Afterwards, the essential oils corresponding to each one of the storage periods (1–7 days, 15–30 days and 100–120 days) were blended and analysed.

A 30 litre stainless steel still and saturated steam (0.5 bar) produced in an electric boiler (ETE, Madrid, Spain) were used during the batch steam distillation tests. The steam flow was 15 kg/h and the extraction duration 30 min per sample distilled. Time was measured from the moment the first drop of distillate fell. The temperature inside the still was kept constant at 98 °C. The hydrolate and the essential oil were separated by density using a glass Florentine flask and the samples were collected in glass flasks. The essential oils were dried over anhydrous sodium sulphate. After filtration they were weighed and stored at 4 °C until further analysis. The oil yield was calculated as a percentage (weight/weight) on a biomass dry basis.

### 4.3. Essential Oil Analysis

The essential oil components were analysed by GC (FID)-MS, using the relative area percentage. The used apparatus were Agilent HP 8890/5977 (GC-MS) and Agilent HP 8890 (GC-FID) (Agilent Technologies, Santa Clara, CA, USA), both equipped with DB-WAX UI fused silica columns (60 m × 0.25 mm inner diameter, film thickness 0.5 µm) and retention time locking. The column temperature program was 50 °C for a duration of 6 min, increase of 2 °C/min to 190 °C, then 4 °C/min increase to 220 °C, which was maintained for 10 min and, finally, 4 °C/min increase to 250 °C, which was maintained for 10 min. The carrier gas was helium at a variable flow rate and a head pressure of 30.75 psi. The injection volume was 0.1 µL and split mode injection (ratio 1:100) was employed. Injector and detector temperatures were 240 °C. GC-MS was performed with the same capillary column, carrier gas and operating conditions described for GC analysis. Mass spectra were taken over the m/z range 33–350 with an ionisation voltage of 70 eV.

The individual components were identified by MS and their identity was confirmed by comparison of their Kovats retention index calculated using co-chromatographed standard hydrocarbons relative to C_6_-C_30_ *n*-alkanes and mass spectra with reference samples and those of the computer libraries (NIST 14, Wiley 10 and Chromessence library built through standards injection) and available data in the literature [41].

### 4.4. Statistical Analysis

Statistical analysis of the data set corresponding to the essential oil yields across the storage times was performed using Statgraphics Centurion XVII.I. A variance analysis (ANOVA) was used to determine statistically significant differences across the storage periods. Statistically significant difference at *p*-value of the F-test below 0.05 was considered.

## 5. Conclusions

The storage of *Cistus ladanifer* L. bales from mechanised harvesting during 100–120 days does not significantly decrease the essential oil yield. Consequently, a mechanised harvesting of this species together with the storage of the plants could be contemplated by the industry to increase the volume of harvested plants and to improve the working conditions during harvesting, compared to the traditional manual harvesting.

On the other hand, although in the different periods tested there was a decrease in the content of the total monoterpene compounds and an increase in the oxygenated sesquiterpenes, mainly during the 100–120 days of storage, no differences were found in the qualitative composition of the essential oils obtained in the three storage periods studied.

## Figures and Tables

**Table 1:** Essential oil yields obtained in the batch steam distillations with stored *Cistus ladanifer*.

Storage Time (Days)	Moisture Content (%, w.m.)	Yield (%, d.b.)	Aver.	Std. dev.	Rel. std. dev.
1	32.0	0.063			
2	30.9	0.082			
4	30.2	0.088			
7	29.8	0.068			
1–7			0.075	0.012	15.5
15	29.7	0.082			
20	28.8	0.055			
30	30.0	0.060			
15–30			0.066	0.014	21.9
100	15.8	0.050			
110	15.2	0.060			
120	15.2	0.047			
100–120			0.052	0.0068	13.0

w.m.: wet matter; d.b.: dry basis; Aver.: average; Std. dev.: standard deviation; Rel. std. dev.: relative standard deviation.

**Table 2:** Main components (≥0.3%) of *Cistus ladanifer* essential oil analysed by GC-MS and GC-FID.

Component	RI_cal_	RI_ref_	0–7 Days Rel. Area (%)	15–30 Days Rel. Area (%)	100–120 Days Rel. Area (%)
Total monoterpene hydrocarbons			60.92	58.23	56.50
α-pinene	1020	1025	49.65	47.42	46.67
Camphene	1062	1069	2.60	2.49	2.56
β-pinene	1108	1110	0.61	0.87	0.56
Sabinene	1121	1122	0.37	0.38	0.47
Verbenene	1125	1124	1.20	0.93	0.47
α-terpinene	1182	1178	0.33	0.41	0.42
Limonene	1203	1198	2.08	1.81	1.52
β-phellandrene	1212	1209	0.61	0.41	0.29
γ-terpinene	1252	1245	0.79	0.87	0.86
*p*-cymene	1278	1279	1.47	1.26	1.33
α-terpinolene	1294	1290	0.21	0.36	0.22
Total oxygenated monoterpenes			10.34	10.37	8.15
1,8-Cineole	1217	1211	0.30	0.26	0.21
2,2,6-Trimethylcyclohexanone	1328	1328	0.46	0.40	0.41
α-campholenal	1498	1496	0.18	0.73	0.16
Isopinocamphone	1562		0.63	0.59	0.42
Pinocarvone	1589	1586	1.04	0.92	0.75
Bornyl acetate	1595	1592	2.71	2.32	2.48
Terpinen-4-ol	1613	1601	0.76	0.54	0.47
Myrtenal	1643	1635	0.38	0.32	0.33
*trans*-pinocarveol	1668	1664	1.56	1.59	0.99
Myrtenyl acetate	1699	1698	0.57	0.53	0.56
Borneol	1704	1701	0.38	0.35	0.28
Total sesquiterpene hydrocarbons			6.54	6.05	6.63
α-ylangene	1497	1490	0.53	0.53	0.60
α-copaene	1506	1492	0.78	0.79	0.81
Alloaromadendrene	1665	1661	1.50	1.13	1.68
Ledene	1704	1704	0.21	0.24	0.37
α-muurolene	1742	1740	0.46	0.51	0.51
δ-cadinene	1777	1772	0.97	1.13	1.11
α-calacorene	1924	1921	0.36	0.42	0.49
Total oxygenated sesquiterpenes			14.57	17.81	18.64
Palustrol	1952	1938	0.13	0.43	0.45
Ledol	2059	2057	2.85	3.41	3.51
Viridiflorol	2113	2104	10.03	11.98	12.50
Spathulenol	2146	2144	0.34	0.39	0.41
T-muurolol	2199	2191	0.49	0.63	0.70
Others			2.02	1.69	2.38
Sclareoloxide	2278		0.34	0.61	0.85
Total identified			94.39	94.15	92.30

RI: retention index relative to C_6_-C_30_ *n-*alkane on DB-WAX column (_cal_: calculated, _ref_: reference, according to Pherobase database [36]); Rel. area: relative peak areas calculated by GC-FID.

## Data Availability

Not applicable.

## Sample Availability

The data leading to the results of this work are available from the authors upon reasonable request.
